# A Micro-Extraction Technique Using a New Digitally Controlled Syringe Combined with UHPLC for Assessment of Urinary Biomarkers of Oxidatively Damaged DNA

**DOI:** 10.1371/journal.pone.0058366

**Published:** 2013-03-06

**Authors:** Berta Mendes, Pedro Silva, Fernando Aveiro, Jorge Pereira, José S. Câmara

**Affiliations:** 1 CQM - Centro de Química da Madeira, Centro de Ciências Exatas e da Engenharia, Universidade da Madeira, Funchal, Portugal; 2 Centro de Ciências Exatas e da Engenharia, Universidade da Madeira, Funchal, Portugal; 3 SESARAM, Hospital Dr. Nélio Mendonça, Funchal, Portugal; Ludwig-Maximilians-University Munich, Germany

## Abstract

The formation of reactive oxygen species (ROS) within cells causes damage to biomolecules, including membrane lipids, DNA, proteins and sugars. An important type of oxidative damage is DNA base hydroxylation which leads to the formation of 8-oxo-7,8-dihydro-2′-deoxyguanosine (8-oxodG) and 5-hydroxymethyluracil (5-HMUra). Measurement of these biomarkers in urine is challenging, due to the low levels of the analytes and the matrix complexity. In order to simultaneously quantify 8-oxodG and 5-HMUra in human urine, a new, reliable and powerful strategy was optimised and validated. It is based on a semi-automatic microextraction by packed sorbent (MEPS) technique, using a new digitally controlled syringe (eVol^®^), to enhance the extraction efficiency of the target metabolites, followed by a fast and sensitive ultrahigh pressure liquid chromatography (UHPLC). The optimal methodological conditions involve loading of 250 µL urine sample (1∶10 dilution) through a C8 sorbent in a MEPS syringe placed in the semi-automatic eVol^®^ syringe followed by elution using 90 µL of 20% methanol in 0.01% formic acid solution. The obtained extract is directly analysed in the UHPLC system using a binary mobile phase composed of aqueous 0.1% formic acid and methanol in the isocratic elution mode (3.5 min total analysis time). The method was validated in terms of selectivity, linearity, limit of detection (LOD), limit of quantification (LOQ), extraction yield, accuracy, precision and matrix effect. Satisfactory results were obtained in terms of linearity (r^2^ > 0.991) within the established concentration range. The LOD varied from 0.00005 to 0.04 µg mL^−1^ and the LOQ from 0.00023 to 0.13 µg mL^−1^. The extraction yields were between 80.1 and 82.2 %, while inter-day precision (*n = *3 days) varied between 4.9 and 7.7 % and intra-day precision between 1.0 and 8.3 %. This approach presents as main advantages the ability to easily collect and store urine samples for further processing and the high sensitivity, reproducibility, and robustness of eVol^®^MEPS combined with UHPLC analysis, thus retrieving a fast and reliable assessment of oxidatively damaged DNA.

## Introduction

**Figure 1:**
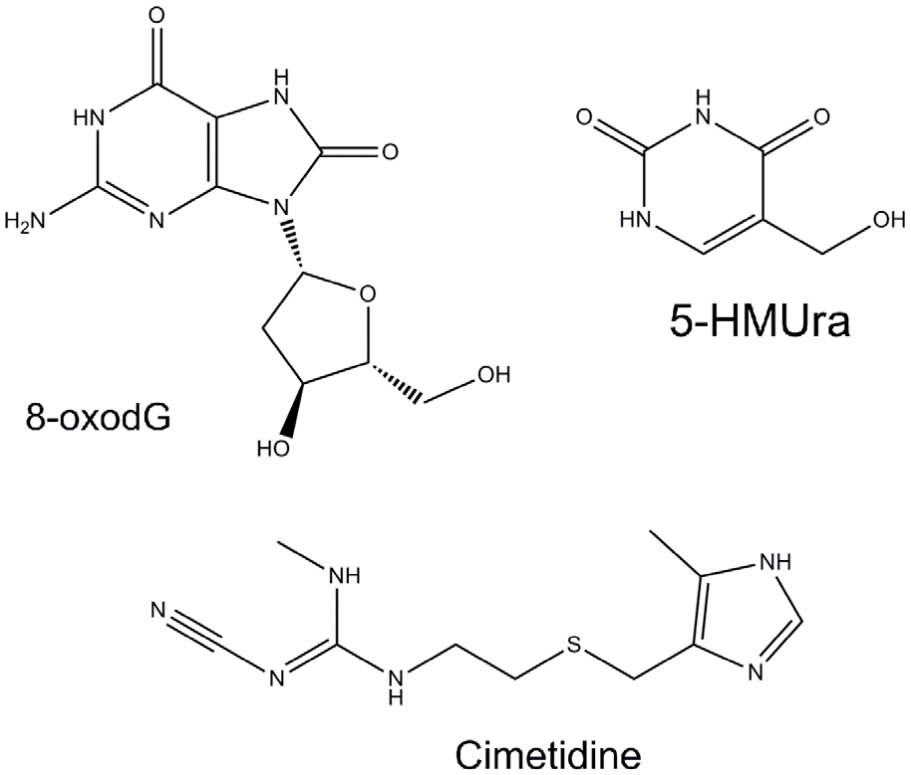
Chemical structures of 8-hydroxy-2′-deoxyguanosine (8-oxodG), 5-hydroxymethyluracil (5-HMUra) and cimetidine (used as internal standard - IS).

Oxidative stress results from an imbalance between the generation of reactive oxygen species (ROS) and antioxidant defences. It occurs when excessive production of ROS overwhelms the antioxidant defence system, when there is any condition affecting the antioxidant defences or a combination of both factors [1], [2]. Oxidative stress is known to cause damage to biomolecules, namely membrane lipids, DNA, proteins and sugars, a condition known as oxidative damage. This damage is particularly harmful to DNA when there are mutations in tumour suppressor genes that are not corrected, triggering critical initial events in carcinogenesis [3]. This attack to DNA generates a wide range of base and sugar modification products that include single- or double-stranded DNA breaks, purine, pyrimidine, or deoxyribose modifications, and DNA cross-links (reviewed in [4]). Over one hundred of such oxidatively modified DNA forms have been characterised in vitro [5], but only about 20 identified *in vivo* [4] and found to be involved in the induction of signal transduction pathways, replication errors and genomic instability and transcription induction or arrest [6]. These results are strongly supported by, for example, the high levels of oxidative lesions in cancer tissues, and reduced cancer incidence in populations with high dietary antioxidant intake [7]. Breast and lung cancers are two particularly prevalent forms of cancer worldwide, being breast cancers, more intimately related to DNA damage repair defects or defects in cell-cycle checkpoints which allow damaged DNA to go unrepaired [8], while in lung cancer, unrepaired DNA damage and genomic instability predominate [9]. Therefore, assessment of DNA damage is very important and this can be performed by quantifying oxidatively modified DNA adducts such as 8-oxodG (reviewed in [10]). 8-oxodG results from the oxidation of guanine at the C8 position, leading to site-specific mutagenesis in bacterial and mammalian cells through G-T transversions that are widely found in mutated oncogenes and tumour suppressor genes [6]. Moreover, elevated levels of oxidative DNA lesions, namely 8-oxodG, have been noted in various tumours, strongly implicating such damage in the etiology of cancer, most probably in the initiation process [11]. Although less studied, 5-HMUra is another important and frequent oxidative DNA lesion and can result from the hydroxylation of thymine, forming the HmU:A mismatched base pair, or hydroxylation followed by deamination of 5-methylcytosine, resulting in HmU:G base pair formation [12], [13]. Therefore, 5-HMUra is also used to assess oxidative damage to DNA [14]. In Figure 1 are represented the structures of both biomarkers.

The extraction methods developed for the analysis of biomarkers of oxidatively modified DNA, generally, includes extractions with solvents, clean-up and further fractionation by liquid–liquid extraction (LLE) [15], or solid phase extraction (SPE) [16], [17]. However, these procedures are quite time-consuming and need a relatively high volume of solvent/sample, which is impractical for the routine analyses. In clinical laboratories the main requirements for sample preparation are celerity, simplicity and miniaturisation of the experimental procedures, especially when using small sample volumes and organic solvents, while maintaining sufficient selectivity, precision and accuracy [18]. Microextraction by packed sorbent (MEPS), as a miniaturised SPE, was a logical extension of that technique for the sample preparation of biological fluids, presenting several advantages. It can handle both small sample and large volumes (from 10 µL up to 1000 µL) and is suitable for normal phases, reversed phases, mixed mode and ion exchangers. Therefore, MEPS can substitute most existing methods using SPE just by scaling down the solvents and sample volumes. This approach is much less expensive because the MEPS syringe can be used several times, up to 100 times or more, while conventional SPE columns and cartridges are used only once. Moreover, its utilisation prior to liquid chromatography is an excellent tool for screening the presence of drugs and metabolites in blood, plasma and urine samples. It was already used to extract a wide range of analytes of interest in biological matrices, namely antiepileptic [19] and psychotropic drugs, local anaesthetics and their metabolites, anticancer drugs and the neurotransmitters dopamine and serotonin (reviewed in [20]).

Current quantification methods for 8-oxodG analysis are mainly immunological (enzyme-linked immune sorbent (ELISA) assays [21], [22]) or chromatographic (HPLC with electrochemical detection (HPLC-ECD) [23], capillary electrophoresis with electrochemical detection (CE-ECD) [24], HPLC coupled to mass spectrometry detection with electrospray ionisation (HPLC-ESI-MS/MS) [25], or gas chromatography coupled with mass spectrometry detection (GC-MS) [24]). In turn, 5-HMUra quantification has been reported only a few times and using a GC-MS approach [13], [26], [27]. The improvement of HPLC technology leads to UHPLC development that very rapidly became a new standard in separation sciences. Very recently, Lam *et al.* (2012) presented a methodology to quantify 8-oxodG in several human fluids using UPLC-MS/MS [28].

The present research study reports, for the first time, to the best of our knowledge, the development and validation of an ultrafast, efficient, sensitive, reliable and high throughput MEPS eVol^®^-based methodology in combination with UHPLC–PDA for the simultaneous determination of two urinary biomarkers of oxidatively damaged DNA, 5-HMUra and 8-oxodG, in cancer (lung and breast) patients and healthy subjects (control). Chromatographic conditions were optimised in order to achieve increased sensitivity and high resolution on the target metabolites while maintaining reduced analysis time (less than 3.50 min). The levels of the metabolites are evaluated and compared in order to determine their potential for cancer diagnosis as a first approach. We then compared the alterations in the oxidatively damaged DNA profiles between the cancer patients and the controls using univariate and multivariate analyses.

## Materials and Methods

### Ethics Statement

All cancer patients and healthy subjects gave their written informed consent for inclusion in the study and the research was approved by the Ethics Committee of the Dr. Nélio Mendonça Hospital (Funchal, Portugal), being done in accordance with the Good Clinical Practice guidelines and with the ethical guidelines of the 1975 Declaration of Helsinki. All data were analysed anonymously throughout the study.

### Subjects and Sample Collection

The subjects were divided into healthy subjects (control group) and cancer (breast and lung) patients. The characterisation of the groups is shown in Table 1.

**Table 1:** The characteristics of subjects (Age, Gender, Smoking habits, Medication).

			Cancer Patients	
		Control	Breast	Lung
**Age**	21–40	7	1	1
	41–60	7	5	6
	61–80	6	4	3
**Gender**	Male	9	–	7
	Female	11	10	3
**Smoking Habits**	Yes	1	2	3
	No	19	8	5
	Ex	–	–	2
**Medication**	Yes	9	8	9
	No	11	2	1

The normal controls (*n = *20, age = 48.7 ± 17.6 y) were selected among the blood donors at the Dr. Nélio Mendonça Hospital and had no clinical history of cancer. The patients (breast cancer *n = *10, age = 57.1 ± 11.7 y; lung cancer, *n = *10, age = 54.8 ± 10.8 y) underwent different diagnostic procedures, such as breast physical examination, mammography and ultrasonography, magnetic resonance imaging and chest X-ray and finally histologically diagnosed with primary cancer by the Haematology–Oncology Unit of the Dr. Nélio Mendonça Hospital.

Each individual (either patient or healthy volunteer) provided a sample of morning urine (after overnight fasting) in a 20 mL sterile PVC container. The samples were immediately frozen at −80°C and kept until being processed.

### Standard Preparation and Urine Samples

Individual standard solutions of oxidatively damaged DNA biomarkers (1000 µg mL^−1^) were prepared in pure water, aliquot in 4 mL vials, and stored at −20°C. Under these conditions they were stable for at least 4 months (as assessed by UHPLC). Working standard solutions containing the oxidative stress biomarkers were prepared daily from the individual stock solutions by diluting them in the synthetic urine (SU, prepared as described by Uppuluri *et al.* [29]).

The ranges of concentrations (*see* Table 2) were selected according to the sensitivity of the UHPLC–PDA towards each biomarker (as the physical-chemical characteristics of each compound affect its analytical signal, higher concentrations had to be used for some compounds in order to be possible their detection). In all measurements (standards and samples) cimetidine was used as internal standard (IS). All samples were analysed in triplicate and their pH previously adjusted to 6 with 0.1 M sodium hydroxide or 0.01 % formic acid and filtered through 0.22 µm membrane PTFE filters.

**Table 2:** Figures of merit of the newly developed eVol^®^-MEPS/UPLC-PDA methodology.

Biomarkers		5-HMUra	8-oxodG
Peak number		1	3
RT (min)		1.05	1.80
λ_max_ a (nm)		215	295
Analytical performance			
Conc. range (µg mL^−1^)		0.0005 – 0.01	0.1–5
Regression equation		*y* = 1277.5*x* + 0.8318	*y* = 0.0724*x* + 0.0052
b r^2^		0.9906	0.9946
LOD^c^ (µg mL^−1^)		0.00005	0.04
LOQ^d^ (µg mL^−1^)		0.00023	0.13
% Matrix effect		80.1	82.2
Fortification level (µg mL^−1^)^e^	LL	0.0005	0.1
	ML	0.0025	1.5
	HL	0.01	5.0
Accuracy (%)^e^	LL	91.0	94.7
	ML	95.5	96.8
	HL	104.7	103.5
Extraction yield (%)^e^	LL	89.4	63.5
	ML	98.5	73.7
	HL	99.9	101.8
Intra-day (*n = 7*) RSD(%)^e^	LL	5.7	8.3
	ML	4.2	4.8
	HL	0.9	2.0
Inter-day (*n = 25*) RSD(%)^e^	LL	7.7	6.1
	ML	7.7	5.8
	HL	6.1	4.9

a Maximum absorbance values obtained in the PDA system detection;

b Correlation coefficient, give an estimating how well the experimental points fit a straight line;

c Limit of detection;

d Limit of quantification. Values obtained from ordinary least-squares regression data.

e Concentration levels used in eVol^®^MEPS/UPLC-PDA validation studies: LL-low level; ML- medium level; HL- high level.

### MEPS Extraction using eVol^®^

The MEPS procedure was carried out with an eVol^®^ semi-automatic syringe (SGE Analytical Science, Melbourne, Australia), consisting of a 500 µL gas-tight syringe with a removable needle.

The syringe was fitted with a BIN containing 4 mg of the sorbent material through which samples and solutions were discharged. A SU sample spiked with known amounts of oxidative stress biomarkers was used to optimize the MEPS procedure. The flow rate during aspiration was limited to 20 µL s^−1^ to prevent cavitation. This also increases analyte/sorbent contact time and extraction efficiency. All optimization procedures were carried out in triplicate. Before each use, the sorbent was conditioned with 100 µL of methanol followed by 0.01 % formic acid. This step activates the sorbent and ensures reproducible retention of the analytes between extractions, decreasing memory effects (carry-over) [20].

### UHPLC-PDA Analysis and Operating Conditions

The analysis of oxidative stress biomarkers were carried out on a Waters Ultra Performance Liquid Chromatographic Acquity system (UPLC, Acquity H-Class) (Milford, MA, USA) combined with a Waters Acquity quaternary solvent manager (QSM), an Acquity sample manager (SM), a column heater, a PDA detector, and a degassing system. The whole configuration was driven by Empower software v2.0 (Waters Corporation). Optimum separation was achieved with a binary mobile phase which consisted of (A) water at 0.01% formic acid, and (B) methanol, with a constant flow rate of 250 µL min^−1^, giving a maximum back pressure of 6.000 psi, which is within the capabilities of the UHPLC. The extracts (2 µL) were injected into the UPLC system, equipped with an Acquity UPLC^TM^ strength silica HSS T3 analytical column (1.8 µm particle size, 2.1×100 mm) protected with an Acquity UPLC^TM^ HSS T3 Van Guard^TM^ Pre-column (Waters, Milford, USA). The column temperature was thermostated at 30°C and the samples were kept at 15°C in the sample manager. The sample analysis was performed with an isocratic flow of 80 % A at 250 µL min^−1^ during 3.50 min followed by a re-equilibration time of 3 min. All solvents and samples were filtered through 0.22 µm PTFE filters (Millipore, Milford, USA), before use. For quantification purposes the PDA detection was conducted by using four distinct channels that were set to the maximum absorbance wavelength of each biomarker, as indicated in Table 2. They were identified by comparing the retention time and spectral characteristics of their peaks.

### Method Validation Design

The eVol^®^MEPS/UHPLC-PDA newly developed method for determination of urinary biomarkers of oxidatively damaged DNA was fully validated in terms of selectivity, linearity, limits of detection (LOD) and quantification (LOQ), inter- and intra-day precision, accuracy, extraction efficiency and matrix effect (Table 2). The selectivity of the method was assessed by the absence of interfering peaks at the elution times of 5-HMUra and 8-oxodG. Method linearity was evaluated by constructing three calibration curves (the peak area_analyte_/peak area_IS_ ratios obtained were plotted against the corresponding standard concentration) for each biomarker using standard solutions prepared in SU from individual stock solutions. It were prepared eight different concentration levels in triplicate for each point (*n* = 9), including zero point, in order to cover the whole working range (Table 2). The zero point (unspiked SU) enables the verification that none of the compounds showed residual level or background signal. The sensitivity of the method was assessed by determining the LOD (the lowest analyte concentration that produces a response detectable above the noise level of the system) and LOQ (the lowest level of analyte that can be accurately and precisely measured) for each compound. LOD and LOQ were calculated with the data generated in the linearity studies, being LOD defined as (a+3S_a/b_) and LOQ as (a+10S_a/b_), where “*a*” represents origin ordinate, “*S_a_*” the origin ordinate variance and “*b*” the slope [30]. These parameters were calculated for each analyte from the standard solutions used to obtain the corresponding calibration curves, using the developed UHPLC method.

Precision is a function of the concentration and describes the closeness of agreement between series of measurements, facilitating comparisons of variability at different concentrations. Method precision was evaluated by spiking a SU at three different concentration levels, corresponding to the low level (LL), medium level (ML) and highest point (HL) of the calibration curve of each biomarker (Table 2). Six replicates (*n* = 6) were performed in the same day to obtain repeatability (intra-day precision). For inter-day precision (intermediate precision) evaluation, the same protocol was followed but six replicates of each level were analysed daily through three different days (*n = *24).

In order to check the accuracy of the proposed method a recovery study was carried out by spiking SU, in triplicate at three concentration levels corresponding to the LL, ML and HL (Table 2), and subjected to the eVol^®^MEPS procedure above (*section 2.4.1*). The recovery values were calculated according to the following formula: *Accuracy = *100×([analyte]_after spiking_ – [analyte]_before spiking_)/[analyte]_added_; where [analyte]_after spiking_ is the analyte concentration measured in spiked urine; [analyte]_before spiking_ is the analyte concentration measured in unspiked urine, and [analyte]_added_ is the nominal concentration of the analyte added to urine. Extraction efficiency (EE) was determined by replicate analysis (*n = *3) of SU spiked with oxidative stress biomarkers at three concentration levels (LL, ML, and HL; *see* Table 2) and submitted to eVol^®^MEPS procedure (C_SU_MEPS_); a second set of different aliquots of SU was submitted to eVol^®^MEPS and the extracts spiked with the 5-HMUra and 8-oxodG biomarkers at LL, ML, and HL concentration levels (C_SU_). The peak area ratio obtained for spiked SU matrix before and after eVol^®^MEPS was used to calculate the corresponding concentration through regression analysis (interpolation of signals in calibration graphs). The matrix effect was evaluated by the percentage of the quotient between the slopes of the standards in blank matrix (SU) and those obtained by spiking urine (standard addition method).

### Statistical Analysis

Significant differences among the three extraction techniques were assessed with a one-way analysis of variance (ANOVA) using a SPSS Program, version 19.0 (SPSS Inc. Headquarters, Chicago IL, USA). Figures and tables were generated using Microsoft Office Excel 2007 (Microsoft Corporation, Redmond, WA, USA).

## Results

### Optimisation of the MEPS Procedure

**Figure 2:**
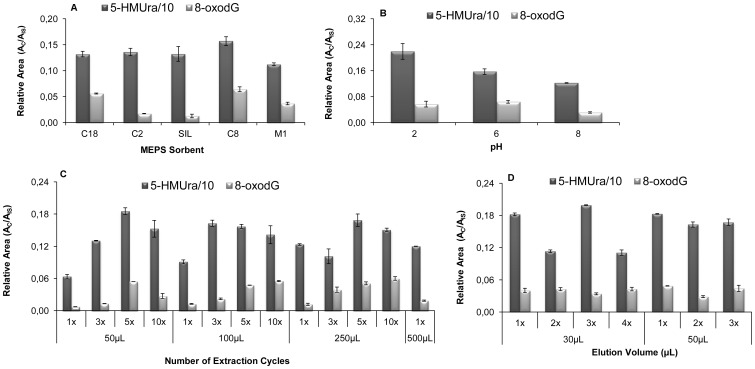
Optimisation results of the eVol^®^MEPS extraction methodology for quantification of oxidatively damaged DNA biomarkers in urine. (A) Comparison the performance characteristics (relative peak areas - A_C_/A_IS_, obtained for each adsorbent) of different MEPS adsorbents for isolation of target DNA damage biomarkers from urine; (B) effect of pH value on the extraction efficiency; (C) influence of number of extraction cycles (extraction-discard) as a function of applied sample volume; (D) effect of elution volume on UHPLC-PDA response. Errors bars show the standard deviation of the mean (*n* = 3).

To optimise the MEPS procedure, important experimental parameters with influence on the extraction performance, namely sorbent type, sample pH, sample volume, and elution conditions, were carefully investigated. Each one of the commercially eVol^®^MEPS sorbents (the silica-based C2, C8, C18, SIL and the mixed-mode M1 (C8/strong cation exchanger (SCX)), was evaluated. The selection of the best sorbent was based on extraction efficiency, determined by the relative peak area, and reproducibility. As shown in Figure 2A, the highest relative peak areas for both biomarkers (5-HMUra and 8-oxodG) were obtained with C8 sorbent. In contrast, the lowest extraction efficiency was obtained by using M1 and SIL sorbents, respectively. C8 sorbent was, therefore, chosen as the best stationary phase and was used for more than 100 extractions without loss of efficiency, as assessed by the UHPLC-PDA data generated.

The influence of pH on the extraction efficiency was evaluated by assaying samples with pH adjusted to 2, 6 and 8 (using 0.1M sodium hydroxide solution or 0.01% formic acid). The results (Figure 2B) showed that pH 2 enables the best results for 5-HMUra, while pH 6 is slightly better for 8-oxodG. However, the peak resolution for both biomarkers is much better at pH 6 (data not shown) and urine pH usually range between 6 and 7 [31]. Therefore, pH 6 was chosen to perform the analytical extraction of the target biomarkers. The effect of the number of extraction cycles (extract–discard) and sample volume on extraction efficiency of the biomarkers was shown in Figure 2C. The best result for 5-HMUra was obtained with five extraction cycles for 50 µL or 250 µL of sample volume, while for 8-oxodG were five extraction cycles for 50 µL and ten extraction cycles for 100 or 250 µL of sample volume. As there was no significant improvement in the two biomarkers extraction efficiency by using higher sample volumes, five extraction cycles of 50 µL sample volume loading were used. This choice of using low sample volume loads several times (5) has additional advantages of extending the lifetime of the MEPS cartridge and minimisation of possible interferences of other urine compounds in the target biomarkers quantification. The elution conditions were also assayed and, as shown in Figure 2D, the extraction efficiency was not significantly affected by the increase in the number or volume of elutions. Therefore, it was chosen to elute the target analytes with three times 30 µL of methanol / 0.01 % formic acid (elution solution).

### Method Validation

The method performance parameters were calculated for each biomarker using concentrations usually found in human urine. The validation parameters are shown in Table 2.

**Figure 3:**
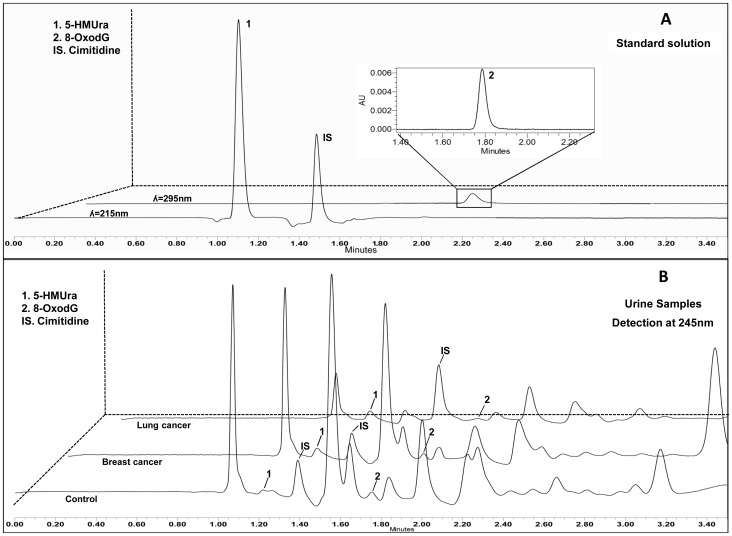
Representative UHPLC-PDA chromatograms of a biomarker standard solution (5-HMUra at 0.0025 µg mL^−**1**^; 8-oxodG at 4 µg mL^−1^) (A), and from urine of control subjects and cancer (lung and breast) patients (B).

The selectivity of the new approach was assessed by the absence of interference in the same chromatographic windows as examined in a solution of standards of both biomarkers and analysis of “blank matrices” (SU). No interfering peaks were observed in the blank chromatograms at the quantification wavelengths (215 and 295 nm for 5-HMUra and 8-oxodG, respectively, see Figure 3).

Linearity of the method was established on spiked urine samples prepared and analysed using the described extraction procedure in the range of 0.005–0.01 µg mL^−1^ for 5-HMUra and 0.1–5 µg mL^−1^ for 8-oxodG (six calibrators evenly distributed, three replicates). The UHPLC–PDA system gave linear response over the studied range of concentrations and the least-squares linear regression analysis of the data provided excellent correlation coefficient values for 5-HMUra (r^2^ > 0.9906) and 8-oxodG (r^2^ > 0.9946). The calibration was performed by using SU spiked with the calibration standards prepared as previously described (Material and Methods). The eVol^®^MEPS/UHPLC–PDA methodology allows very low detection (0.00005 µg mL^−1^ and 0.04 µg mL^−1^) and quantification limits (0.00023 and 0.13 µg mL^−1^) for 5-HMUra and 8-oxodG, respectively. This sensitivity is sufficient to quantify the target biomarkers in biological fluids.

The precision values are satisfactory, with relative standard deviation (RSD) values lower than 8.5 % for each measured analyte at all spiking levels (Table 2). The intra-day precision at the three different levels ranged from 1.0 % (5-HMUra at HL concentration) to 8.3 % (8-oxodG at LL concentration), while the inter-day precision varied from 5.0 % (8-oxodG at HL concentration) to 7.7 % (5-HMUra at ML concentration).

The accuracy of the method was determined according to the equation presented in method validation design section. The mean accuracies obtained for the target biomarkers (*n = *6) at each fortification level are listed in Table 2. At high concentrations, the results were satisfactory and ranged between 95.5 % and 104.7 %, and at low concentrations the recovery was slightly lower (91.0 %). The results showed that the absolute extraction yield increased slightly from low concentration levels (89.4 % for 5-HMUra, and 63.5 % for 8-oxodG) to medium (98.5 % 5-HMUra, and 73.7 % for 8-oxodG) and high concentration levels (99.9 % for 5-HMUra, and 91.0 % for 8-oxodG). To evaluate the impact of the matrix on the target analytes, the slopes obtained in the calibration with matrix-matched standards were compared with those obtained with solvent-based standards, calculating matrix/solvent slope ratios for each of both metabolites (Table 2). We consider that the matrix effect could be ignored if the matrix/solvent slope ratio values were in the range of 85 % – 110 %, below that limit, a matrix suppression effect is observed and above 110% there is matrix enhancement [32]. Based on the results obtained (80 % and 82 % for 5-HMUra and 8-oxodG, respectively) a moderate matrix effect was observed and, therefore, matrix-matched calibration solutions were used for quantification purposes, in order to compensate the referred errors.

### Quantification of 8-oxodG and 5-HMUra by eVol^®^MEPS / UHPLC-PDA

The assays were carried out using SU as the development method matrix. After method validation, and in order to demonstrate its applicability, a total of forty urine samples (lung and breast cancer patients and healthy subjects) were analysed in triplicate. Both biomarkers were identified by the retention time and UV spectra obtained at the maximum absorbance wavelength (λ_max = _215 nm and 295 nm for 5-HMUra and 8-oxodG, respectively). A typical chromatogram of control, lung and breast cancer patients urine obtained through eVol^®^MEPS / UHPLC-PDA developed methodology, is shown in Figure 3.

As can be observed, excellent peak shape and resolution were achieved with minimal interference from other components of the urine matrix. Moreover, all urine profiles are quite similar, despite the clinical condition of the donor, except for the concentrations of each biomarker analysed.

**Figure 4:**
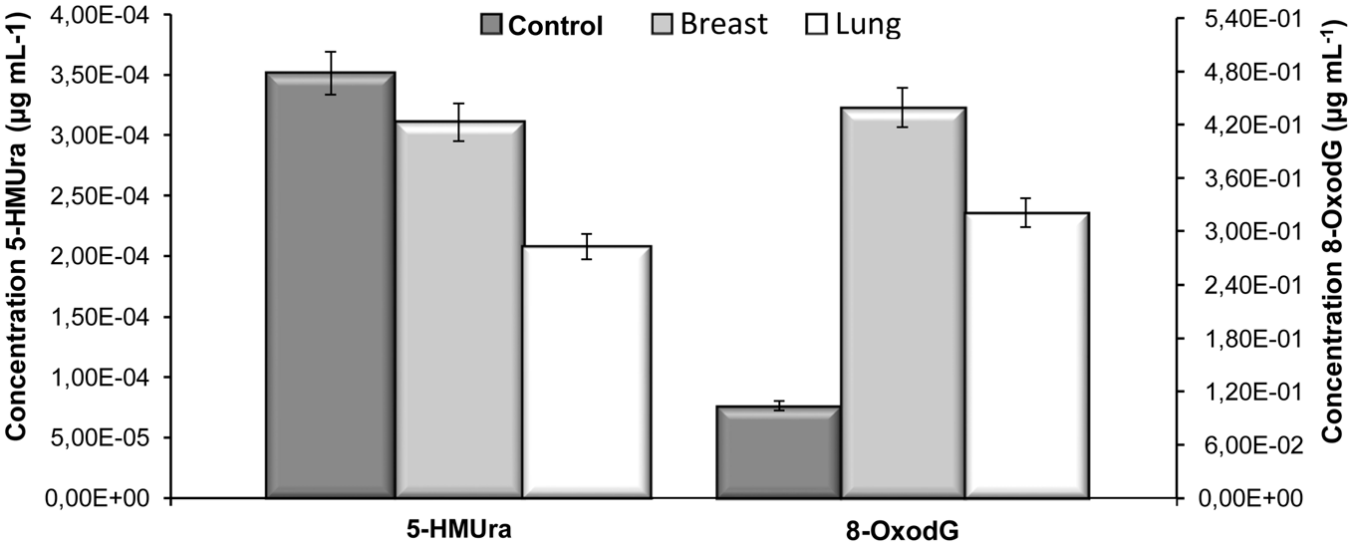
Total concentration of oxidatively damaged DNA investigated biomarkers in normal controls, and in breast and lung cancer patients.

With respect to the controls, twenty urine samples (*n = *20), were analysed, and the mean values obtained of 2.9×10^−4^ ± 3.0 (RSD (%)) µg mL^−1^ and 1.9×10^−1^ ± 4.4 (RSD (%)) µg mL^−1^ for 5-HMUra and 8-oxodG, respectively. Regarding cancer patients, ten lung cancer patients (*n = *10) and ten breast cancer patients (*n = *10) were analysed. For lung cancer patients, the mean values obtained were 2.3×10^−4^ ± 3.2 (RSD (%)) µg mL^−1^ and 2.6×10^−1^ ± 3.9 (RSD (%)) µg mL^−1^ for 5-HMUra and 8-oxodG, respectively. With respect to the breast cancer patients, the mean concentrations were 3.1×10^−4^ ± 2.6 (RSD (%)) µg mL^−1^ and 4.4×10^−1^ ± 5.2 (RSD (%)) µg mL^−1^ for 5-HMUra and 8-oxodG, respectively. These results can be more easily compared by viewing Figure 4.

## Discussion

The optimization of MEPS procedure involved several factors and conditions described in the previous section. The best cumulative sorbent for both biomarkers, C8, is in agreement with the fact that C2–C8 phases are more suitable for lipophilic analytes (non-polar) and polymeric phases such as polystyrene–divinylbenzene, while mixed mode phases (anion–cation exchange mode) are more suitable for polar analytes such as acidic and basic compounds [33]. Moreover, C8 sorbents present extra properties, allowing secondary interactions with polar groups from the analyte. However it should be noted that 8-oxodG is much more selective in the interaction with the stationary phases, with C2 and SIL retrieving very low responses when compared with remain sorbents. In turn 5-HMUra interacts more favourably with all sorbents (Figure 2). In MEPS, the retention of the analytes in the sorbent phase is affected by the number of extraction cycles performed and the speed applied. Experimentally, the multiple extraction cycles can be performed using the same aliquot (draw–eject in the same vial) or by drawing up from the aliquot and discarding in waste (extract–discard). This last option was selected in this study. Figure 2C shows that the competition for active adsorption sites of the C8 sorbent increased slightly until five extraction cycles. However, the volume of sample loading is not directly proportional to the increase of relative area.

Regarding the method performance, the results obtained demonstrated a good linearity for both biomarkers investigated, within the tested concentration range for the whole extraction and determination method. In general, acceptable recoveries and precision were obtained for 5-HMUra and 8-oxodG at three different spiked levels. Furthermore, optimised UHPLC-PDA offers good sensibility and selectivity for the target analytes.

The obtained results for the extraction yields showed low extraction efficiency at low concentration level (LL) for 5-HMUra and 8-oxodG, than those obtained with the middle (ML) and high (HL) fortification levels. The reason for this may be due to the fact that the surface chemistry of the sorbent can be altered by interfering compounds from urine and, therefore, sorption properties can change [34]. This effect is more pronounced at low concentrations of the analyte when the analyte/matrix ratio is very low. The same behaviour was observed for the accuracy measurements. Despite this, the results show that eVol^®^MEPS/UHPLC-PDA is a good methodology to quantify 5-HMUra and 8-oxodG in human urine. Unfortunately, there are no reference ranges for 8-oxodG and 5-HMUra in healthy or diseased individuals [35] and we can only compare our results with the ones reported by others using similar methodologies. In this respect, Harri *et al.* (2007) [15], using HPLC-MS/MS, reported lower values than ours for 8-oxodG (8-oxodG concentrations ranging from 0.16 to 16.48 µg L-1, LOD = 0.005 µg L^−1^ and LOQ = 0.16 µg L^−1^). Regarding 5-HMUra, we only find one study [26] referring to the concentration of 121 pmol mL^−1^ for this biomarker, but using HPLC-GC-MS, which makes it very difficult to compare methodologies.

Forty human urine samples (20 normal controls; 10 lung cancer patients and 10 breast cancer patients) were used in the study. As shown in Figure 3, the separation of the standard mixture of the biomarkers of DNA oxidation is very fast, being achieved in less than 3.50 min. The chromatograms present a good peak shape and resolution was achieved for both compounds with no interference from urine matrix (Figure 3). The chromatograms obtained for control individuals and for breast and lung cancer patients showed quite similar profiles (Figure 3), since the concentrations of both biomarkers in the samples are very low to present any significant difference in the overall chromatogram. Accordingly, with these results, the concentration of 5-HMUra does not present significant differences between controls subjects and breast patients showing a slight decrease in lung cancer patients. In turn, 8-oxodG presents more pronounced differences between control, the lowest levels, lung cancer patients, intermediate levels which are almost twice the control levels, and breast cancer patients, that present values that are three times higher than controls.

There are few studies showing that the levels of urinary 8-oxodG increases in breast and lung cancer patients [36], [37], [38], but to our knowledge, our study is the first one reporting the simultaneous quantification of 8-oxodG and 5-HMUra, using a MEPS-UHPLC-PDA approach, which is much more reliable than the referred studies using ELISA assays. Overall, the combination of eVol^®^MEPS together with quick UHPLC-PDA system, proved to be an improved strategy, with excellent recoveries, sensitivity, and repeatability, making it possible to use, as a rapid approach, to analyse the biomarkers present in human urine. Moreover, the combination of eVol^®^ and MEPS offers improvements in workflow and accuracy of the extraction process, because, unlike conventional SPE columns, the MEPS sorbent bed can be easily integrated into a liquid-handling syringe, as the semiautomatic syringe eVol^®^, allowing for low void volume sample manipulations and the full automation of the sample processing, extraction and injection steps. Recently, Lam et al. [28] quantified 8-oxodG in several human matrices using a SPE-LC-MS approach. The methodology we are now proposing can be considered an improvement to Lam’s report because the use of MEPS instead of SPE certain would benefit their work. Furthermore, the methodology would be an alternative to assessing oxidative damage to DNA when MS detection is not available, as the MEPS-UHPLC approach presents enough sensitivity and is much less expensive, and is more environment-friendly than the previous one.

In summary, a robust, rapid and fully validated procedure is described for the detection and high-throughput quantification of oxidatively-damaged DNA biomarkers in human (healthy subjects and cancer patients) urine samples, using MEPS/UHPLC-PDA. This method has shown to be linear within the adopted ranges for both biomarkers, and presented adequate accuracy and precision. Furthermore, the procedure can be useful for those laboratories performing routine urine analysis in the field of both clinical and medicinal chemistry.
